# A Diagnosis Fit for a Queen: Crowned Dens Syndrome

**DOI:** 10.5811/cpcem.19473

**Published:** 2024-11-18

**Authors:** Erin N. Dankert Eggum, Sara A. Hevesi, Benjamin J. Sandefur

**Affiliations:** Mayo Clinic, Department of Emergency Medicine, Rochester, Minnesota

**Keywords:** crowned dens syndrome, neck pain, pseudogout

## Abstract

**Case Presentation:**

We describe a case of an elderly female patient with a history of pseudogout who presented to the emergency department with atraumatic neck pain, fever, and malaise, who was found to have crowned dens syndrome on computed tomography imaging.

**Discussion:**

It is important that emergency physicians consider crowned dens syndrome in elderly patients presenting with neck pain and signs of inflammation to ensure timely diagnosis, treatment, and to minimize unnecessary invasive testing.

## CASE PRESENTATION

A 76-year-old female presented to the emergency department with five days of posterior neck pain and generalized weakness. The pain was sharp and began when she extended her arms to catch a ball. Movement exacerbated the pain, and it was unresponsive to over-the-counter analgesic medications. The pain radiated into both upper extremities. She reported three days of subjective fever and malaise. There was no numbness or weakness. Medical history was pertinent for cervical spine osteoarthritis and pseudogout.

On examination, the patient was well appearing. Vital signs included heart rate 90 beats per minute, blood pressure 166/62 millimeters of mercury, respiratory rate 19 breaths per minute, and temperature 37.2 degrees Celsius. She was sitting upright and tense in bed; however, she refused to move her neck due to pain. She had a normal neurologic exam, including normal strength and sensation, no meningismus, and no cervical spine tenderness to palpation. Laboratory studies revealed an elevated C-reactive protein at 158.4 milligrams per liter (mg/L) (reference range less than 5.0 mg/L), erythrocyte sedimentation rate at 105 millimeters per hour (mm/hr) (0–29 mm/hr), and a normal leukocyte count at 9.5×10^9^ per liter (L) (3.4–9.6×10^9^/L). Computed tomography (CT) angiogram of the head and neck was obtained, and the initial report identified no acute findings. Magnetic resonance imaging of the cervical spine was obtained and revealed no discitis, osteomyelitis, or epidural abscess.

**Image 1:**
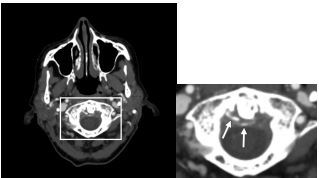
Computed tomography angiogram of the head and neck, axial view, showing calcification of the periodontoid ligament (arrows) from calcium pyrophosphate dihydrate crystal deposition surrounding the odontoid process, creating a crown appearance.

**Image 2:**
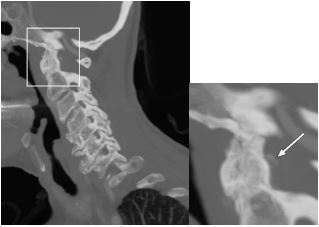
Computed tomography angiogram of the head and neck, sagittal view, showing calcification of the periodontoid ligament (arrow) surrounding the odontoid process.

The patient remained unable to move her neck despite intravenous (IV) analgesics. Upon further review of the CT images, calcification of the periodontoid ligaments was identified, which can be seen in crowned dens syndrome (Images 1 and 2). During hospitalization, the patient received IV and oral steroids, declined therapy with nonsteroidal anti-inflammatory drugs (NSAIDs), and demonstrated clinical improvement over a 12-hour period with conservative measures.

## DISCUSSION

Crowned dens syndrome (CDS), first described by Bouvet et al in 1985, is characterized by a painful inflammatory condition resulting from calcium pyrophosphate dihydrate (CPPD) or hydroxyapatite crystalline deposition in the cervical spine ligaments.^1^ Despite its clinical significance, awareness of CDS among front-line physicians remains limited. Crowned dens syndrome may account for up to 1.9% of acute neck pain presentations in outpatient settings.^1^,^2^ It may mimic meningitis, giant cell arteritis, discitis, rheumatoid arthritis, polymyalgia rheumatica, and epidural abscess. Misdiagnosis can result in the patient undergoing invasive procedures such as lumbar puncture or temporal artery biopsy.^2^,^3^

Patients are typically female (60%) with an average age of 71 years. Crowned dens syndrome is more common in patients with pseudogout of peripheral joints.^2^ Symptoms include localized pain at the base of the skull resulting in neck stiffness. There is often systemic evidence of inflammation, including fever (80.4%) and elevated inflammatory markers (88.3%).^2^,^3^ The frequent occurrence of fever in CDS aligns with its classification as an inflammatory, crystalline deposition disease, similar to other conditions within this category.^4^ Non-enhanced CT is the gold standard for diagnosis.^2^,^5^ A crown-like appearance around the odontoid process on coronal views is observed, representing calcification from crystalline deposits of CPPD or hydroxyapatite.^1^,^3^ Magnetic resonance imaging is not sensitive in identifying calcification but is superior in excluding spinal cord compression.^6^ Treatment includes NSAIDs; however, oral colchicine or corticosteroids may be used if the patient has contraindications to NSAIDs.

**CPC-EM Capsule**

What do we already know about this clinical entity?

*Although information is sparse on crowned dens syndrome (CDS), we know it frequently presents in patients with a history of pseudogout.*

What is the major impact of the image(s)?

*The images will assist clinicians in considering this diagnosis for their patients.*

How might this improve emergency medicine practice?

*Emergency physicians should consider CDS in elderly patients presenting with neck pain and signs of inflammation to minimize unnecessary invasive testing.*

It is important that emergency physicians consider CDS in elderly patients presenting with atraumatic neck pain, elevated inflammatory markers, and fever to ensure timely diagnosis and treatment, and to minimize unnecessary invasive testing.
